# Protecting public’s wellbeing against COVID-19 infodemic: The role of trust in information sources and rapid dissemination and transparency of information over time

**DOI:** 10.3389/fpubh.2023.1142230

**Published:** 2023-04-17

**Authors:** Yingnan Zhou, Airong Zhang, Xiaoliu Liu, Xuyun Tan, Ruikai Miao, Yan Zhang, Junxiu Wang

**Affiliations:** ^1^School of Sociology and Ethnology, University of Chinese Academy of Social Sciences, Beijing, China; ^2^Health and Biosecurity, CSIRO, Brisbane, QLD, Australia; ^3^School of Mental Health, Wenzhou Medical University, Wenzhou, China; ^4^Institute of Sociology, Chinese Academy of Social Sciences, Beijing, China; ^5^School of Psychology, Inner Mongolia Normal University, Hohhot, China; ^6^Faculty of Ideological and Political Education and Moral Education, Beijing Institute of Education, Beijing, China; ^7^Mental Health Education Center, Shijiazhuang Tiedao University, Shijiazhuang, China

**Keywords:** psychological stress, trust, media sources, information dissemination, perceived safety, wellbeing, information transparency

## Abstract

**Objectives:**

This study examined how trust in the information about COVID-19 from social media and official media as well as how the information was disseminated affect public’s wellbeing directly and indirectly through perceived safety over time.

**Methods:**

Two online surveys were conducted in China, with the first survey (Time1, *N* = 22,718) being at the early stage of the pandemic outbreak and the second one (Time 2, *N* = 2,901) two and a half years later during the zero-COVID policy lockdown period. Key measured variables include trust in official media and social media, perceived rapid dissemination and transparency of COVID-19-related information, perceived safety, and emotional responses toward the pandemic. Data analysis includes descriptive statistical analysis, independent samples *t*-test, Pearson correlations, and structural equation modeling.

**Results:**

Trust in official media, perceived rapid dissemination and transparency of COVID-19-related information, perceived safety, as well as positive emotional response toward COVID-19 increased over time, while trust in social media and depressive response decreased over time. Trust in social media and official media played different roles in affecting public’s wellbeing over time. Trust in social media was positively associated with depressive emotions and negatively associated with positive emotion directly and indirectly through decreased perceived safety at Time 1. However, the negative effect of trust in social media on public’s wellbeing was largely decreased at Time 2. In contrast, trust in official media was linked to reduced depressive response and increased positive response directly and indirectly through perceived safety at both times. Rapid dissemination and transparency of COVID-19 information contributed to enhanced trust in official media at both times.

**Conclusion:**

The findings highlight the important role of fostering public trust in official media through rapid dissemination and transparency of information in mitigating the negative impact of COVID-19 infodemic on public’s wellbeing over time.

## 1. Introduction

COVID-19 has been constantly evolving since its outbreak in early 2020. At the beginning, there was limited scientific understanding and knowledge about the coronavirus. Due to the unknown nature of the novel virus, misinformation and rumors were widely spread across social media platforms, which instilled a strong sense of out-controlled crisis (1–6). Over 2 years into the pandemic, scientific understanding of COVID-19 has been advanced, and vaccines have been developed. Protective measures such as wearing mask, sanitizing hands, and keeping social distance have been commonly adopted in daily life, which is regarded as a “new normal.” While the virus has been constantly mutating, so were rumors and misinformation, especially regarding the COVID-19 vaccines. For example, exaggeration of side effects (e.g., infertility, chronic illness, mental illness) as well as distrust in vaccine development (e.g., crucial trials skipped) were widespread on social media, leading to vaccine hesitancy (7–13). Meanwhile, the preventive measures and COVID-19-related policies taken by governments were also changing over time and different from country to country. While most of countries have reopened by early to mid-2022, strict lockdown and COVID-zero policy were still in place in China. Such misinformation and differences in government policies have kept sending confusing message to the public. This situation highlights the remarkable characteristics of the concurrence of virology and virality of COVID-19, where fast virus spreading is coupled with rapidly spreading of information and misinformation (14). Precisely as WHO Director-General Dr. Ghebreyesus pointed out, “We’re not just fighting an epidemic; we are fighting an infodemic” (15).

Extensive empirical studies from different countries have demonstrated that a broad range of rumors and misinformation about COVID-19 spread across social media, which negatively impacted public’s wellbeing and posited challenge for pandemic control (1–7). Research has shown that trust in COVID-19 information from social media was negatively linked to accurate knowledge about COVID-19 (16), positively linked to beliefs in COVID-19 myths and false information (17) as well as vaccine hesitancy (5, 18, 19). Moreover, rumors and misinformation fueled fears and led to psychological distress among the public over the course of COVID-19 pandemic (20–29). Frequently using social media as an information source for COVID-19 was significantly related to poorer psychological wellbeing (28, 30–32). Moreover, erroneous, inconsistent, unverified, and often conflicting news and messages led to uncertainty, which caused intense stress to the public (33). Emerging research indicates that perceived vulnerability to COVID-19 mediated the relationship between exposure to COVID-19 news and depressive symptoms (34). In addition, when people used social media to obtain COVID-19-related information, their perceived risk of being infected heightened as the level of concern increased (35). In turn, higher risk perception and lack of perceived safety toward COVID-19 led to increased anxiety and depressive symptoms (36–39). Those findings suggest that the conflicting information and uncertainty on social media made people feel unsafe as it is not clear how to protect oneself. This led to fear and stress, and hence, impacted wellbeing. However, how trust in social media affect public’s wellbeing during COVID-19 pandemic, and the mediating role of perceived safety are not yet directly examined. Informed by the research reviewed above, we hypothesized that:

*H1*: Trust in COVID-19-related information from social media was negatively associated with positive emotional response and positively associated with depressive emotional response toward COVID-19.

*H2*: Perceived safety mediates the relationship between trust in social media with positive and negative responses toward COVID-19, respectively.

To minimize public fear and confusion caused by social media, transparency and rapid dissemination of information by government agencies has been suggested crucial (40–42). The role of transparency and trust was also demonstrated in managing public fear and panic in SARS outbreak in Singapore (43) as well as during other outbreaks including Ebola in West Africa and MERS-CoV in South Korea (44). Indeed, timely, accurate and transparent information from officials is foundational for the public to implement protective measures, mitigate the negative impact of the pandemic, and to reduce psychological distress in the crisis (45, 46). The satisfaction with governments’ communication about COVID-19 was linked to public trust in government (47, 48). These findings suggest that transparency and rapid dissemination of information about COVID-19 is the key factors to build public trust in official media. Therefore, we hypothesized:

*H3*: Perceived rapid dissemination and transparency of the information about COVID-19 are positively related to trust in official media.

With respect to how trust in official media would affect public’s wellbeing, existing literature pointed to different directions. Some studies indicated that official media in some countries applied a fear-based communication strategy (e.g., showing realistic pictures and giving direct information on COVID-19 death statistics) and suppressed scientific debate to persuade public to adhere to recommended health behaviors such as wearing mask, practicing social distance, and getting vaccinations (49–52). Such fear-inducing approach can increase levels of perceived threat, cause psychological distress, and affect wellbeing among the public (51, 53–55). In this case, trust in official media would negatively affect public’s wellbeing through decreasing perceived safety from being infected. Meanwhile, other studies suggested the opposite. These studies found trust in the government and obtaining information from official media reduced perceived risk toward COVID-19, mitigated mental distress, and improved psychological wellbeing among the public (35, 56–58). These findings suggested that receiving information from trusted and authoritative source would give people certainty and efficacy, hence, increasing perceived safety and enhancing mental wellbeing. In summary, the research findings reviewed above indicate that trust in COVID-19-related information from official media could either positively, or negatively affect public’s wellbeing and that perceived safety might play a mediating role. Hence, we proposed that trust in official media was significantly related to public’s wellbeing both directly and indirectly through perceived safety (Hypotheses 4 and 5), but we left the direction of the relationships (i.e., positive or negative) open.

*H4*: Trust in COVID-19-related information from official media was significantly (either positively or negatively) associated with positive and depressive emotional responses toward COVID-19 respectively.

*H5*: Perceived safety mediates the relationship between trust in official media with positive and negative responses toward COVID-19 respectively.

### The present study

The present study aimed to investigate how trust in the information about COVID-19 from official media and social media affect public’s wellbeing (i.e., positive response and depressive response) through perceived safety, and how the dissemination of information impact public trust in official media both at the early stage of COVID-19 outbreak and 2 years later in China. To our best knowledge, this is the first study to examine the impacts of trust in media sources on public’s wellbeing toward COVID-19 over time. The insights developed through this study will help policy makers and health intervention initiatives develop targeted strategies to address the mental health challenges presented by the COVID-19 pandemic and protect public’s wellbeing.

**Figure 1:**
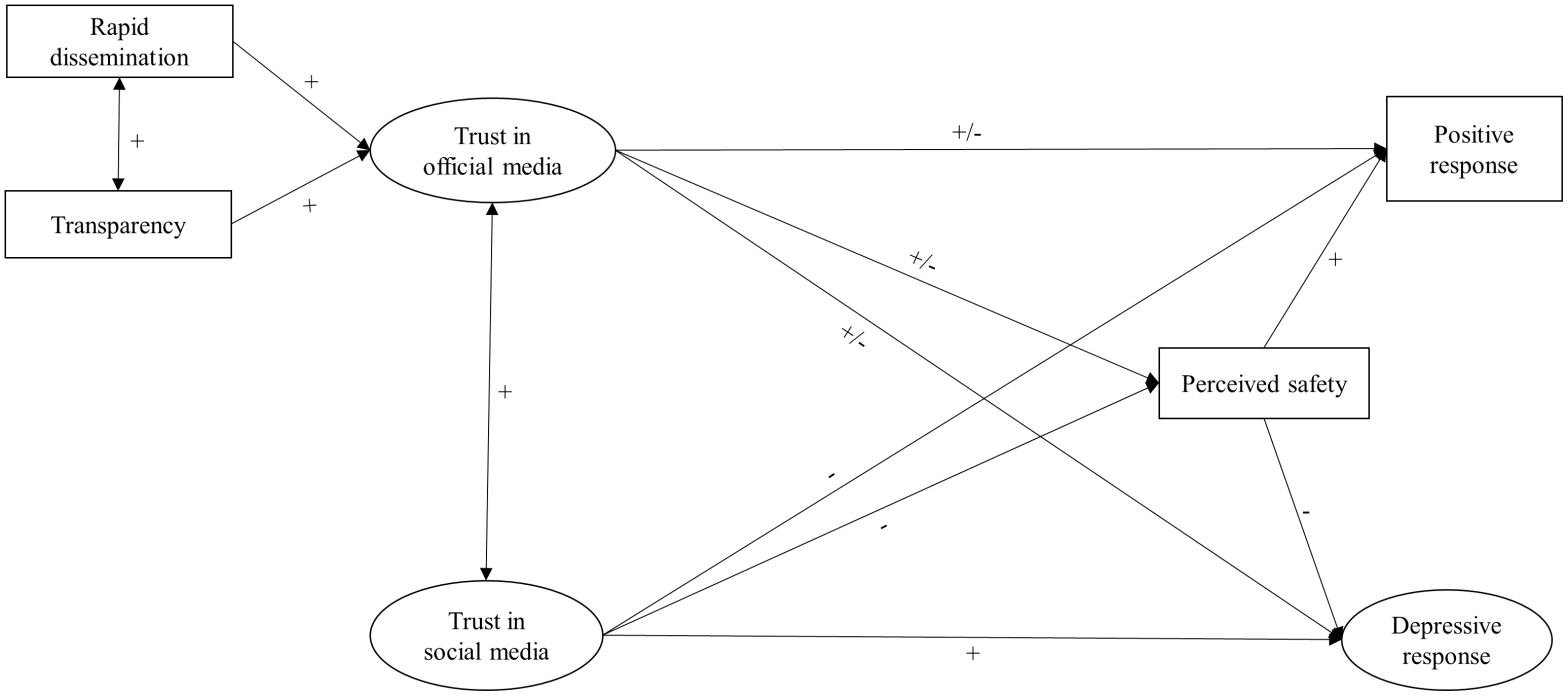
An integrative model to predict emotional responses toward the COVID-19.

Figure 1 presents a path model which summarizes the hypotheses proposed above. In this model, we propose that trust in COVID-19 information received from social media was negatively associated with positive emotional response and positively associated with depressive emotional response toward COVID-19 both directly and indirectly through decreased perceived safety (H1-H2); that perceived transparency and rapid dissemination of COVID-19-related information are positively related to trust in official media (H3). In turn, trust in official media was either positively or negatively associated with positive and depressive response toward COVID-19 both directly and indirectly through perceived safety (H4, H5).

Though the scientific understanding of COVID-19 has been advanced over 2 years into the pandemic, the “infodemic” wasn’t over. Rumors and misinformation about the virus and vaccine were still widespread across social media (8). In addition, the mental health symptoms were still quite prevalent among public in the “new normal” era (59). Therefore, it’s important to examine the mechanism of trust in media sources affect public’s wellbeing over time. The path framework we proposed allows the examination of how the key factors affect public’s wellbeing both at the early stage of COVID-19 outbreak and post COVID-19 era and also allows to make comparisons of the changes in effects. The developed insights on what has changed over time will inform policy makers to adjust the risk communication strategies accordingly.

## 2. Materials and methods

### 2.1. Procedure and participants

National online surveys in China were conducted at the early stage of COVID-19 outbreak and 2 years later. Time 1 survey was carried out between 24-Jan to 10-Feb, 2020, which was right after China’s official announcement of COVID-19 outbreak (on January 20, 2020) and deployed lockdown measures (on January 23, 2020). Time 2 survey was conducted between 21-Apr to 4-May, 2022, when Delta and Omicron variants were widely spread around the world and COVID-zero policy was still in place in China (60). The study was conducted in compliance with the ethical standards specified in the Ethical Principles of Psychologists and Code of Conduct by the American Psychological Association (61) and in 1964 Helsinki declaration and its later amendments (62). Two private research survey companies (Intell-vision for Time 1, ePanel for Time 2) were engaged to recruit participants and conduct data collection through convenance sampling. The survey link was sent to users of the online survey platforms of the two companies. After presenting a brief description of the study, participants were informed that no personal identifiable information would be collected and that their survey results would remain confidential. Participants were further informed that their participation was voluntary and that they could withdraw from the survey at any time without penalty. Participants were asked to click ‘I agree’ button if they consent to participate in the survey. Participants who completed the survey were paid a small fee for their participation. The collected data was completely anonymous, and the research team was the only party has access to the data.

Table 1 presents participants’ demographic information for both Time 1 and Time 2.

**Table 1:** The sample characteristics.

Variables	Values	
	Time 1 (*N* = 22,702)	Time 2 (*N* = 2,901)
Age (years)	28.41 (SD = 9.90/Range = 18–70)	31.77 (SD = 8.05/Range = 18–69)
Gender		
Male	10,866 (47.9%)	1,274 (43.9%)
Female	11,836 (52.1%)	1,627 (56.1%)
Education		
Junior high school and below (Year 9 or below)	796 (3.5%)	16 (0.6%)
Senior high school (Year 12)	3,287 (14.5%)	137 (4.7%)
College certificate	3,514 (15.5%)	416 (14.3%)
Bachelor’s degree	10,952 (48.2%)	2,115 (72.9%)
Postgraduate	4,153 (18.3%)	217 (7.5%)

### 2.2. Measures

#### 2.2.1. Trust in official media

At the early stage of COVID-19 outbreak, the official news reached the public largely through television news and it was also available online in China. The TV news report is in the format of news from central government first and followed by news from local government. Hence, at Time 1, trust in official media was measured by asking participants to indicate how trustworthy the information on the Coronavirus outbreak from central government-owned media and local government-owned media, respectively, on a 4-point scale (1 = not trustworthy at all, 4 = very trustworthy; *α* = 0.75). While 2 years later, community social workers also became important information sources. They conveyed official information on COVID-19 to the public and implemented preventive and control measures at community level. Therefore, at Time 2, trust in official media was measured by asking participants to indicate how trustworthy the information on COVID-19 from central government-owned media, local government-owned media, and community social workers, respectively, on a 5-point scale (1 = not trustworthy at all, 5 = very trustworthy; *α* = 0.75). To compare the change between Time 1 and Time 2, the score of trust in official media at Time 1 was transformed to a 5-point scale by using the following formula (63, 64):


$$X_{1}={\left(4/3\right)}^{*}\;X-\left(1/3\right)$$

Here:

*X*1: Transformed score of trust in official media (on a 5-point scale).

*X*: the original score of trust in official media (on a 4-point scale).

#### 2.2.2. Trust in social media

In the beginning of COVID-19 outbreak, Weibo and WeChat were the most popular social media platforms in China for the spread of information about COVID-19. Besides, acquaintances were also important information sources during the pandemic. Hence, at Time 1, trust in social media was measured by asking participants to indicate how trustworthy the information on the Coronavirus outbreak from Weibo influencers, WeChat influencers, and acquaintances, respectively, on a 4-point scale (1 = not trustworthy at all, 4 = very trustworthy; *α* = 0.77). As time passed by, the general netizens became more and more important in information transmission. Therefore, at Time 2, trust in social media was measured by asking participants to indicate how trustworthy the information on COVID-19 from internet influencers, general netizens, and acquaintances, respectively, on a 5-point scale (1 = not trustworthy at all, 5 = very trustworthy; *α* = 0.68). The Cronbach’s alpha for trust in social media at Time 2 is a bit lower than the widely considered desirable value of 0.70 (65, 66). However, a low number of items could lead to a low value of Cronbach’s alpha (65). Since there were only 3 items in this scale, an alpha value of 0.68 is acceptable (67, 68). To examine the difference between Time 1 and Time 2, the score of trust in social media at Time 1 was also transformed to a 5-point scale by using the formula described above (63, 64).

#### 2.2.3. Rapid dissemination, transparency, and perceived safety

Rapid dissemination was measured with: “So far, do you think the dissemination of information about Coronavirus is rapid?” (1 = very delayed, 4 = very rapid). Transparency was measured with: “So far, how transparent do you think the information on the Coronavirus outbreak is?” (1 = very low, 4 = very high). Perceived safety was measured with: “Thinking about Coronavirus, how safe do you feel from being infected?” (1 = not safe at all, 4 = very safe).

#### 2.2.4. Emotional responses

The measurement of emotional responses toward COVID-19 outbreak was adapted from the Florida Shock Anxiety Scale (69, 70). The Florida Shock Anxiety Scale (FSAS) was developed to measure patients’ psychological distress caused by the threat and fear of potential implantable cardioverter defibrillators (ICD) shock. The COVID-19 pandemic has instilled people with a sense of fear of being infected with the virus. The potential infection may happen but is not certain, which makes people feel worried, scared, and angry. This psychological distress is very similar to that elicited from the anticipation of experiencing ICD shock. Hence, we adapted this scale to measure the emotional responses toward COVID-19. Participants were asked to rate their feelings toward COVID-19 outbreak using a 5-point scale (1 = not at all, 5 = very much) on the adjectives describing positive response (optimistic) and depressive response (worried, scared, sad, and angry; *α* = 0.80 at Time 1, *α* = 0.81 at Time 2).

### 2.3. Data analysis

SPSS version 22.0 with AMOS version 24.0 was used for the data analysis. Descriptive statistical analysis, independent samples *t*-test, and Pearson correlations were conducted first. To examine the hypothesized model (Figure 1), A two-stage structural equation modeling approach was conducted (71–77). The analyses for the model at both Time 1 and Time 2 utilized a covariance matrix as input and used maximum likelihood estimation. The goodness of fit of the model was assessed using the comparative fit index (CFI), the Non-Normed Fit Index (NNFI), Goodness-of-fit statistic (GFI), and root mean square error of approximation (RMSEA). A satisfactory fit is suggested by CFI > 0.90, NNFI > 0.90, GFI > 0.90, and Standardized RMSEA < 0.08 (72).

## 3. Results

### 3.1. Changes in measured variables over time

Table 2 presents the means and standard deviations of measured variables at both survey times and independent samples *t*-test results between the two time points. On average, participants displayed sound trust in official media both at Time 1 (*M* = 3.94, SD = 0.84) and Time 2 (*M* = 4.17, SD = 0.67), which were significantly higher than trust in social media at both times (Time 1, *M* = 3.07, SD = 0.86, Time 2, *M* = 3.04, SD = 0.67); *t* (22701) = 131.58, *p* < 0.001 and *t* (2900) = 72.87, *p* < 0.001, respectively. Moreover, trust in official media at Time 2 was significantly higher than Time 1 [*t* (4161.46) = −17.18, *p* < 0.001], while trust in social media at Time 2 was significantly lower than Time 1 [*t* (4225.96) = 2.50, *p* < 0.05]. The results indicated that trust in official media largely increased over time, while trust in social media decreased over time.

**Table 2:** Descriptive statistics and independent samples *t*-test results for measured variables.

	*M* (SD)		*t*	df	Cohen’ *d*
	Time 1 (*N* = 22,702)	Time 2 (*N* = 2,901)			
Trust in official media	3.94 (0.84)	4.17 (0.67)	−17.18***	4161.46	−0.28
Trust in social media	3.07 (0.86)	3.04 (0.67)	2.50*	4225.96	0.04
Rapid dissemination	2.75 (0.87)	3.19 (0.64)	−33.40***	4394.35	−0.52
Transparency	2.75 (0.78)	3.08 (0.71)	−23.31***	3866.79	−0.43
Perceived safety	2.80 (0.68)	2.89 (0.65)	−7.22***	3760.10	−0.13
Positive response	3.07 (1.27)	3.33 (0.94)	−13.41***	4405.12	−0.21
Depressive response	3.22 (1.00)	3.09 (0.87)	7.41***	3951.96	0.13

***p < 0.001, *p < 0.05. Trust in official media and trust in social media were measured on a 5-point scale (1 = not trustworthy at all, 5 = very trustworthy). Rapid dissemination was measured on a 4-point scale (1 = very delayed, 4 = very rapid). Transparency was measured on a 4-point scale (1 = very low, 4 = very high). Perceived safety was measured on a 4-point scale (1 = not safe at all, 4 = very safe). Positive response and depressive response were measured on a 5-point scale (1 = not at all, 5 = very much).

The dissemination of information about the Coronavirus was regarded on average less rapid (*M* = 2.75, SD = 0.87) and transparent (*M* = 2.75, SD = 0.78) at Time 1. However, both measures were significantly improved at Time 2 (rapid dissemination: *M* = 3.19, SD = 0.64, transparency: *M* = 3.08, SD = 0.71); Rapid dissemination: *t* (4394.35) = −33.40, *p* < 0.001; Transparency: *t* (3866.79) = −23.31, *p* < 0.001. Perceived safety from being infected with the Coronavirus also enhanced from Time 1(*M* = 2.80, SD = 0.68) to Time 2 (*M* = 2.89, SD = 0.65), *t* (3760.10) = −7.22, *p* < 0.001. At last, positive emotional response toward COVID-19 increased over time (Time 1, *M* = 3.07, SD = 1.27, Time 2, *M* = 3.33, SD = 0.94); *t* (4405.12) = −13.41, *p* < 0.001, while depressive response decreased over time (Time 1, *M* = 3.22, *SD* = 1.00, Time 2, *M* = 3.09, *SD* = 0.87); *t* (3951.96) = 7.41, *p* < 0.001.

Table 3 presents Pearson correlations between the measured variables at both survey times. Positive response was positively related to trust in official media and social media as well as rapid dissemination, transparency, and perceived safety both at Time 1 and Time 2, while depressive response was negatively associated with these variables (except for trust in social media at Time 1, which was not significantly correlated to depressive response). In addition, trust in official media and social media, rapid dissemination, transparency, and perceived safety were positively correlated to each other at both survey times (except for trust in social media and perceived safety at Time 2, which was not significantly correlated). Finally, positive response and depressive response was negatively related at both survey times.

**Table 3:** Pearson correlations between the measured variables at Time 1 and Time 2.

Variables	1		2		3		4		5		6	
	Time 1	Time 2	Time 1	Time 2	Time 1	Time 2	Time 1	Time 2	Time 1	Time 2	Time 1	Time 2
1. Trust in official media	1.00	1.00										
2. Trust in social media	0.33^***^	0.22^***^	1.00	1.00								
3. Rapid dissemination	0.46^***^	0.48^***^	0.26^***^	0.12^***^	1.00	1.00						
4. Transparency	0.49^***^	0.52^***^	0.28^***^	0.16^***^	0.69^***^	0.68^***^	1.00	1.00				
5. Perceived safety	0.31^***^	0.14^***^	0.22^***^	0.03	0.35^***^	0.14^***^	0.37^***^	0.16^***^	1.00	1.00		
6. Positive response	0.28^***^	0.26^***^	0.18^***^	0.12^***^	0.36^***^	0.22^***^	0.36^***^	0.27^***^	0.31^***^	0.23^***^	1.00	1.00
7. Depressive response	−0.18^***^	−0.19^***^	0.01	−0.04^*^	−0.24^***^	−0.21^***^	−0.23^***^	−0.24^***^	−0.28^***^	−0.25^***^	−0.20^***^	−0.38^***^

^***^p < 0.001, ^*^p < 0.05. Trust in official media and trust in social media were measured on a 5-point scale (1 = not trustworthy at all, 5 = very trustworthy). Rapid dissemination was measured on a 4-point scale (1 = very delayed, 4 = very rapid). Transparency was measured on a 4-point scale (1 = very low, 4 = very high). Perceived safety was measured on a 4-point scale (1 = not safe at all, 4 = very safe). Positive response and depressive response were measured on a 5-point scale (1 = not at all, 5 = very much).

### 3.2. The relationship among information dissemination, trust in media sources, perceived safety, and emotional responses over time

A two-stage structural equation modeling approach was conducted (71–77) to examine the hypothesized model. In this approach, the measurement model, which specifies the relationships between the latent constructs and the observed measures, was tested first *via* confirmatory factor analysis (CFA); followed by the structural model, which specifies the relationships among independent, dependent, and mediating variables. In addition, the bias-corrected bootstrap method was carried out to test the indirect effects. 5,000 bootstrapped samples were generated to approximate the confidence interval (CI) of the indirect effects both at Time 1 and Time 2. A 95% CI without zero indicates statistical significance. Furthermore, following the practice of previous studies (78, 79), the structural model was tested for robustness by changing the sample range.

#### 3.2.1. Confirmatory factor analysis (CFA) for the measurement model

Confirmatory factor analysis (CFA) was conducted to examine the measurement model both at Time 1 and Time 2. The measurement model was supported by the model fit indexes at both survey times: Time 1, CFI = 0.97, NNFI = 0.96, GFI = 0.98, and RMSEA = 0.06; Time 2, CFI = 0.93, NNFI = 0.90, GFI = 0.96, and RMSEA = 0.08.

Furthermore, the convergent and discriminant validity of the measurement model were assessed at both times. The convergent validity was evaluated by using standardized factor loadings, composite reliability (CR) and the average variance extracted (AVE) (see Table 4). All items loaded significantly on their respective constructs, with the standardized factor loadings ranging from 0.50 to 0.85, reaching the criterion of 0.50 or above (74). The CR values ranged from 0.68 to 0.81, meeting an acceptable criterion of 0.60 (74). The AVE values ranged from 0.42 to 0.68, reaching the criterion of 0.36 or above (77). These results provided evidence of satisfactory convergent validity. Discriminant validity was assessed by comparing AVE with the squared correlation between constructs. The squared correlations between constructs at both times ranged from 0.00 to 0.20, which were all much lower than AVE values, indicating that the measurement model has satisfactory discriminant validity (73–75).

**Table 4:** The standardized factor loadings, composite reliability (CR), and average variance extracted (AVE) of each construct in measurement model at Time 1 and Time 2.

Construct	Time 1			Time 2		
	Item	Standardized factor loading	CR	AVE	Item	Standardized factor loading	CR	AVE
Trust in official media	Central government-owned media	0.60	0.80	0.68	Central government-owned media	0.74	0.76	0.52
	Local government-owned media	1.00			Local government-owned media	0.84		
					Community social workers	0.56		
Trust in social media	WeChat influencers	0.82	0.79	0.56	Internet influencers	0.73	0.68	0.42
	Weibo influencers	0.85			General netizens	0.70		
	Acquaintances	0.54			Acquaintances	0.50		
Depressive response	Worried	0.62	0.80	0.51	Worried	0.66	0.81	0.52
	Scared	0.77			Scared	0.75		
	Sad	0.79			Sad	0.79		
	Angry	0.66			Angry	0.68		

CR: Composite reliability, AVE: Average variance extracted.

These results suggested that the measurement model is of sufficient quality to examine the structural model.

#### 3.2.2. Pathway analysis for the structural model

Our hypothesized model (Figure 1) specified rapid dissemination and transparency of information as exogenous predictors of trust in official media, both trust in official media and social media as exogenous predictors of perceived safety. Perceived safety, in turn, was identified as a predictor of positive response and depressive response. Moreover, trust in official media and trust in social media also served as exogenous predictors of positive response and depressive response. In this model, trust in official media, trust in social media, and depressive response were latent variables presented using ellipses, while rapid dissemination, transparency, positive response (optimistic) and perceived safety were observed variables presented using rectangles.

The model fit indices suggest that the model provided good fit for the data at both times: Time 1, CFI = 0.94, NNFI = 0.92, GFI = 0.96, and RMSEA = 0.07; Time 2, CFI = 0.92, NNFI = 0.90, GFI = 0.95, and RMSEA = 0.07.

**Figure 2:**
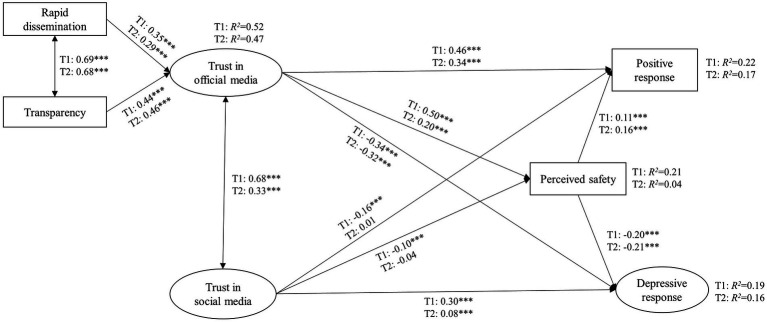
The relationship among information dissemination, trust in media sources, perceived safety, and emotional responses over time.

Figure 2 presents the standardized parameter estimates for the model at both Time 1 (T1) and Time 2 (T2). Table 5 presents the direct, indirect, and total effects of trust in media sources on public’s wellbeing at both Time 1 (T1) and Time 2 (T2).

**Table 5:** The direct, indirect, and total effects of trust in media sources on public’s wellbeing at Time 1 and Time 2.

Paths	Time 1		Time 2	
	Standardized effect	95% CI	Standardized effect	95% CI
Direct effects				
Trust in official media → positive response	0.46^***^	(0.436, 0.479)	0.34^***^	(0.289, 0.396)
Trust in official media → depressive response	−0.34^***^	(−0.360, −0.312)	−0.32^***^	(−0.373, −0.255)
Trust in social media → positive response	−0.16^***^	(−0.181, −0.133)	0.01	(−0.046, 0.065)
Trust in social media → depressive response	0.30^***^	(0.281, 0.328)	0.08^**^	(0.020, 0.150)
Indirect effects				
Trust in official media → perceived safety → positive response	0.06^***^	(0.048, 0.063)	0.03^***^	(0.023, 0.044)
Trust in official media → perceived safety → depressive response	−0.10^***^	(−0.109, −0.092)	−0.04^***^	(−0.057, −0.032)
Trust in social media → perceived safety → positive response	−0.01^***^	(−0.015, −0.008)	−0.01	(−0.015, 0.002)
Trust in social media → perceived safety → depressive response	0.02^***^	(0.016, 0.026)	0.01	(−0.003, 0.019)
Total effects				
Trust in official media → positive response	0.51^***^	(0.494, 0.532)	0.38^***^	(0.321, 0.427)
Trust in official media → depressive response	−0.44^***^	(−0.458, −0.415)	−0.36^***^	(−0.414, −0.298)
Trust in social media → positive response	−0.17^***^	(−0.194, −0.143)	0.00	(−0.054, 0.061)
Trust in social media → depressive response	0.33^***^	(0.300, 0.350)	0.09^**^	(0.026, 0.160)

^***^p < 0.001, ^**^p < 0.01.

First, trust in social media was negatively related to positive response at Time 1 (*β* = −0.16, *p* < 0.001) and positively associated with depressive response both at Time 1(*β* = 0.30, *p* < 0.001) and Time 2 (*β* = 0.08, *p* < 0.001), such that the more people trusted the information about the Coronavirus received in social media, the less they felt optimistic and the more they felt depressive toward the pandemic, especially in the beginning of COVID-19 outbreak. Since trust in social media was no longer significantly related to positive response at Time 2 (*β* = 0.01, *p* = 0.684), Hypothesis 1 was fully supported at Time 1 and was partially supported at Time 2. Moreover, Trust in social media was negatively associated with perceived safety at Time 1 (*β* = −0.10, *p* < 0.001), but not significantly associated with perceived safety at Time 2 (*β* = −0.04, *p* = 0.096). In turn, perceived safety was positively related to positive response (Time 1, *β* = 0.11, *p* < 0.001; Time 2, *β* = 0.16, *p* < 0.001) and negatively linked to depressive response (Time 1, *β* = −0.20, *p* < 0.001; Time 2, *β* = −0.21, *p* < 0.001) at both survey times, suggesting that the safer people felt, the more they were optimistic and the less they were depressed. These results indicated that perceived safety served as a mediator between trust in social media and emotional responses toward COVID-19 at Time 1 but not at Time 2. Thus, Hypothesis 2 was only supported at Time 1.

Second, trust in official media was strongly associated with rapid dissemination (Time 1, *β* = 0.35, *p* < 0.001; Time 2, *β* = 0.29, *p* < 0.001) and transparency (Time 1, *β* = 0.44, *p* < 0.001; Time 2, *β* = 0.46, *p* < 0.001) over time, such that the more people believed information dissemination as rapid and transparent, the more they trusted official media both at the early stage of COVID-19 outbreak and 2 years later. Thus, Hypothesis 3 was supported at both survey times.

Third, trust in official media was positively related to positive response (Time 1, *β* = 0.46, *p* < 0.001; Time 2, *β* = 0.34, *p* < 0.001) and was negatively associated with depressive response (Time 1, *β* = −0.34, *p* < 0.001; Time 2, *β* = −0.32, *p* < 0.001) over time, such that the more people trusted the information about the Coronavirus given by official media, the more they responded optimistically and the less they felt depressively toward the pandemic. Hence, the results provided support for a positive relationship between trust in official media and public’s wellbeing of Hypothesis 4 at both survey times. Furthermore, trust in official media was positively associated with perceived safety at both times (Time 1, *β* = 0.50, *p* < 0.001; Time 2, *β* = 0.20, *p* < 0.001), such that the more people trusted the information about the Coronavirus given by official media, the more they felt safe from being infected. In turn, the safer people felt, the more they felt optimistic and the less they felt depressed. That is, perceived safety mediated the relationship between trust in official media and emotional responses toward COVID-19 both at Time 1 and Time 2. Thus, Hypothesis 5 was supported by a positive mediating effect of perceived safety between trust in official media and public’s wellbeing at both survey times.

The robustness of the structural model was tested by changing the sample range (78, 79). To examine if the structural model only held due to high trust in media sources, we removed a portion of the sample with high trust scores (> 4 out of a possible 5) either in official media or social media. The structural model still held after changing the sample range. And all significant coefficients in the structural model remain significant in robustness check. These results suggest that our findings are relatively robust.

## 4. Discussion

The present research applied a longitudinal approach to examine how trust in media sources affect public’s wellbeing through perceived safety and how the dissemination of information contributes to increased public trust in official media during the course of COVID-19 pandemic.

The results of the present study suggest that the public had more trust in the information about COVID-19 from the official media outlets than from the social media both at the early stage of the pandemic outbreak and 2 years later. The comparatively higher trust in official media is likely due to that the official media represents the voice of the government and is regarded as highly reliable during a pandemic (80, 81). In addition, trust in official media was significantly increased over a two-year period, which is opposite to research findings from Europe and the USA showing trust in official media decreased both in short-term (82) and in long-term (83, 84) during the COVID-19 pandemic. In contrast, trust in social media was slightly decreased two years after COVID-19 outbreak.

Public perceptions of rapid dissemination and transparency regarding information about the Coronavirus also increased over time, which is likely due to the open and transparent risk communication implemented by governments. During COVID-19 pandemic, the Chinese government disclosed real-time data in detail on confirmed, suspected, and cured cases, as well as deaths across the country. It also issued national action plans and released authoritative interpretations of the coronavirus to mitigate public panic and doubts (85). Moreover, public’s wellbeing was significantly improved over the 2 years period, which is in line with research findings from UK (86) and the USA (59).

While the social media were flooded with information and sensational news about COVID-19, public’s trust in them was low. However, trust in social media played a dominant role in contributing to increased depressive symptoms in the early stage of COVID pandemic. The negative impact of trust in social media was largely reduced over time. In contrast, trust in the information from official media was higher, and it played an influential role in contributing to enhanced positive response and decreased depressive symptoms both at the beginning of the pandemic and over 2 years later. While existing literature points to both positive and negative directions regarding how trust in official media would affect mental wellbeing during COVID-19 pandemic (51–58), the present study provides evidence for a positive effect of trust in COVID-19-related information from official media on public’s wellbeing in Chinese context over time. The findings suggest that enhancing public trust in information from official media will be an effective approach to fight against the so called COVID-19 infodemic and protect public’s wellbeing. This has significant implications for public health measures to combat the pandemic of social media panic. To effectively minimize the negative impact of social media on public mental health, health authorities need to rapidly detect and respond to misinformation and rumors in social media.

The present research demonstrated that trust in official media was positively correlated with rapid dissemination and transparency of the information about COVID-19 over time. Hence, fostering and maintaining public’s trust requires rapid dissemination and transparency of information. The trust-building function of transparency revealed in the present study is in line with literature on the general relationship between transparency and public trust (43, 48, 87, 88). Research on infectious disease found that public trust in government and public health authorities as information source influences public perceived risk and their responses to the threat (47, 88–91). The present study further shows that rapid dissemination of information and transparency works hand in hand. These findings suggest that government and health authorities need to rapidly disseminate information and update the outbreak through various platforms including their social media accounts to accommodate all segments of the population. The information needs to be transparent, even though communicating uncertainty and a lack of knowledge in the case of the novel COVID-19 can be unsettling. Otherwise, the absence of official information creates a rich breeding ground for misinformation and rumors in social media, which can further exacerbate the fear caused by the objectively life-threatening nature of the coronavirus. A trusted official media based on transparency and rapid dissemination of COVID-19-related information can keep the public informed and enable them to develop a sense of agency through knowing how to manage the risks.

While the present study has shed light on the negative impacts of trust in social media sources on wellbeing, future research needs to unpack the complexity of social media. The information in social media is diverse and sometimes contradicting. In addition, the information may come from a wide range of sources including people sharing information acquired from official sources (48). Thus, how trust in social media affect public’s wellbeing may depend on the contents and sources. For example, a literature review has shown that viewing stressful content about COVID-19 outbreak on social media was linked to poor psychological outcomes, while viewing motivational and heroic speech, knowledge of COVID-19, and entertaining contents was related to positive psychological wellbeing (45). To unpack the complexity of trust in social media, future research needs to tease apart the information source and contents on social media. The insights will help policy makers and health authorities develop targeted strategy to harness the benefits of social media and mitigate the negative impacts. Moreover, to fully utilize the protective role of trust in official media, an in-depth examination of what key aspects of pandemic related information important for the public is needed. Such insights would inform a more targeted strategy for rapid dissemination. Noticeably, though trust in official media can protect public’s wellbeing against COVID-19 infodemic, this does not mean all the information given by official media is the absolute truth. Scientific understanding of COVID-19 is evolving constantly, such that what qualifies as misinformation might be subjective to new scientific discoveries (6). In addition, fear-based communication strategies may raise public adherence to health recommendations for COVID-19, but such strategies might negatively affect public’s wellbeing (51, 53, 55, 92–94). Future research can unpack the contents and approaches adopted by official media to identify effective communication strategies in conveying information efficiently while protecting public’s wellbeing. At last, although the current study took a longitudinal approach (95), it’s not a follow-up study with the same participants. Future research needs to follow up the same participant sample to further verify the impact of trust in media sources on public’s wellbeing over time.

In summary, the present study has empirically and longitudinally demonstrated that the COVID-19 infodemic can have serious consequence for public’s wellbeing. Especially, trust in the information about COVID-19 in social media was associated with stronger depressive response at the beginning of pandemic. However, trust in official media can mitigate this negative impact. More importantly, the rapid dissemination and transparency of information regarding the virus can enhance public trust in the information from official media outlets. The findings highlight that, to protect public’s wellbeing against COVID-19 infodemic, government and health authorities need to rapidly disseminate information and be transparent even though communicating uncertainty and unknowns can be unsettling. Otherwise, the absence of official information creates a rich breeding ground for misinformation and rumors in social media, which has huge consequence for public’s wellbeing, especially at the early stage of the pandemic.

## Data availability statement

The raw data supporting the conclusions of this article will be made available by the authors, without undue reservation.

## Ethics statement

The studies involving human participants were reviewed and approved by The Academic Committee of Institute of Sociology, Chinese Academy of Social Sciences (CASS). Written informed consent for participation was not required for this study in accordance with the national legislation and the institutional requirements.

## Author contributions

JW, YiZ, and AZ conceived and designed the study. JW, XL, and XT contributed to data collection. YiZ analyzed the data. YiZ, AZ, RM, XT, and XL wrote the first draft of the manuscript. YiZ, AZ, and YaZ revised the manuscript. All authors contributed to the article and approved the submitted version.

## Funding

The study was funded by Key Projects of Philosophy and Social Sciences Research, Ministry of Education of the People’s Republic of China (Award number: 21JZD038) and China Scholarship Council (CSC Award Number: 202004920045).

## Conflict of interest

The authors declare that the research was conducted in the absence of any commercial or financial relationships that could be construed as a potential conflict of interest.

## Publisher’s note

All claims expressed in this article are solely those of the authors and do not necessarily represent those of their affiliated organizations, or those of the publisher, the editors and the reviewers. Any product that may be evaluated in this article, or claim that may be made by its manufacturer, is not guaranteed or endorsed by the publisher.
